# Species-specific identification of adulteration in cooked mutton *Rista* (a Kashmiri *Wazwan* cuisine product) with beef and buffalo meat through multiplex polymerase chain reaction

**DOI:** 10.14202/vetworld.2016.226-230

**Published:** 2016-03-03

**Authors:** M. Mansoor Bhat, Mir Salahuddin, Imtiyaz A. Mantoo, Sheikh Adil, Henna Jalal, M. Ashraf Pal

**Affiliations:** 1 Division of Livestock Products Technology, Sher-e-Kashmir University of Agricultural Sciences & Technology of Kashmir, Shuhama, Alusteng, Srinagar - 190 006, Jammu and Kashmir, India; 2 Division of Livestock Production and Management, Sher-e-Kashmir University of Agricultural Sciences & Technology of Kashmir, Shuhama, Alusteng, Srinagar - 190 006, Jammu and Kashmir, India

**Keywords:** meat adulteration, meat species identification, mitochondrial DNA, multiplex polymerase chain reaction

## Abstract

**Aim:**

Meat adulteration is a serious problem in the meat industry and needs to be tackled to ensure the authenticity of meat products and protect the consumers from being the victims. In view of such likely problem in indigenous meat products of Kashmiri cuisine (*Wazwan*), the present work was performed to study the detection of beef and buffalo meat in cooked mutton *Rista* by mitochondrial DNA (mtDNA) based multiplex polymerase chain reaction (PCR) method under laboratory conditions.

**Materials and Methods:**

Three experimental trials were conducted wherein the products were prepared from pure mutton, beef and buffalo meat, and their admixtures in the ratios of 60:20:20, 80:10:10, 90:05:05 and 98:01:01, respectively.

**Results:**

The primers used in the study amplified the *cyt b* gene fragments of sizes 124 bp, 472 bp and 585 bp for buffalo, cattle and sheep, respectively. It was possible to detect cattle and buffalo meat at the level of 1% in the mixed meat cooked *Rista*. The multiplex PCR successfully amplified *cyt b* gene fragments of mtDNA of the target species and thus produced characteristic band pattern for each species. The band intensities of cattle and buffalo in the mixed meat *Rista* progressively decreased corresponding to their decreasing level from 20% to 1%. Processing, cooking (moist heating) and non-meat formulation ingredients had no effect on detection of meat species adulteration.

**Conclusion:**

The multiplex PCR procedure standardized and developed in this study is simple, efficient, sensitive, reliable and highly specific for detecting falsification of cooked mutton product with beef and buffalo meat up to 1% level.

## Introduction

The first case of fraudulent substitution was recorded in 13^th^ century A.D. at Florence in Italy [1], and this fraudulent adulteration of costly meat with cheap meat is a common practice observed throughout the world. The ability to detect less desirable or objectionable species in meat products is important not only for economic, health, religious, and ethical reasons; but also to ensure fair trade and compliance with legislation [2].

Meat species identification using enzyme-linked immunosorbent assays [3] and protein profiles [4] have been used but in recent years, molecular authentication methodologies based on polymerase chain reaction (PCR) amplification have been developed and successfully applied for species authentication in meat products [5]. The advantages of DNA-based analysis are manifold. First is the ubiquity of DNA from all cell type of an individual contains identical genetic information independent of the origin of the samples. Second, the information content of DNA is a more abundant compared to proteins due to the degeneracy of the genetic codes. Third, DNA is a rather stable molecule that renders the extraction and analysis of DNA from many different types of samples feasible [6]. PCR analysis of species-specific mitochondrial DNA (mtDNA) sequences is the most common method currently used for identification of meat species in food [7-12] and animal feedstuffs [13-15]. Of late, the PCR assays have been employed for the identification of species origin of meat and meat products targeting genomic and mtDNA [16-19]. The detection of species origin of meat employing species-specific primer pairs was employed for authentication of mammalian and poultry species [17,20]. Species-specific PCR assay was developed for quick and authentic identification of chicken, beef and buffalo meat, even in heat processed admixed meat products containing the non-targeted species [21].

*Wazwan* is a multi-course meal in Kashmiri cuisine that is being served at various restaurants and marriage ceremonies with *Rista* as a famous course prepared in red chilli powder and other spices in a very special manner. The present study was thus performed to study the species-specific detection of low-cost beef and buffalo meat adulteration in cooked mutton *Rista* emulsion using multiplex PCR of *cyt b* gene fragments under laboratory conditions.

## Materials and Methods

### Ethical approval

Ethical approval was not necessary as all meat samples were procured from the open market.

### Raw materials

Hot boned mutton, beef and buffalo meat from leg portion of the respective dressed carcass were procured from the open market, packaged in properly labeled low density polythene (LDPE) bags and transported to the Division of Livestock Products Technology, Sher-e-Kashmir University of Agricultural Sciences and Technology of Kashmir (SKUAST-K) for preparation of the product.

### Preparation of *Rista*

The product was prepared according to the standardized processing schedule (Table-1) and recipe of Samoon (1988) with slight modifications. The basic formulation for *Rista*, prepared under various controls, *viz*. mutton (C_M_), beef (C_B_) and carabeef (C_C_) as well as treatments, viz. the admixture of three meats, respectively, in percentages of 60:20:20 (T_1_), 80:10:10 (T_2_), 90:5:5 (T_3_) and 98:1:1 (T_4_), is presented in Table-2. Further, the standardized recipe is presented in Table-2.

**Table-1:** Standardized recipe for *Rista*.

Name of ingredients	Quantity (g)
Raw meatballs	500
Water	1000
Hydrogenated vegetable oil	62.50
Turmeric powder	12.50
Red chili extract	125
Large cardamom	1.25
Small cardamom	0.50
Cinnamon	1.75
Cloves	0.25
Dried ginger powder	2.00
Garlic paste	4.00
Fried leek paste	25
Common salt	5.00

**Table-2:** Proportions of meat and fat used in the formulation for *Rista*.

Percent ingredients	Controls and treatments						

	C_S_*	C_C_*	C_B_*	T_1_*	T_2_*	T_3_*	T_4_*
Mutton	80	-	-	48 (60)	64 (80)	72 (90)	78.40 (98)
Beef	-	80	-	16 (20)	8 (10)	4 (5.0)	0.80 (1.0)
Buffalo meat	-	-	80	16 (20)	8 (10)	4 (5.0)	0.80 (1.0)
Mutton fat	20	-	-	12 (60)	16 (80)	18 (90)	19.60 (98)
Beef fat	-	20	-	4 (20)	2 (10)	1 (5.0)	0.20 (1.0)
Buffalo meat fat	-	-	20	4 (20)	2 (10)	1 (5.0)	0.20 (1.0)
Total	100	100	100	100	100	100	100

* C_S_, C_C_ and C_B_ indicates pure meat *Rista* of mutton, beef and buffalo meat, respectively.

* T_1_, T_2_, T_3_ and T_4_ indicates mixed meat *Rista* with mutton, beef and buffalo meat as 60:20:20, 80:10:10, 90:05:05 and 98:01:01, respectively

### *Rista* processing and sampling

The cooked form of the product of mutton, beef and buffalo meat were prepared according to the standardized processing schedule and recipe [22] with slight modifications. In accordance with the formulation (Table-2), weighed portions of minced meat and fat from each species were taken for the desired meat component for the treatments (admixtures) after thorough mixing. Random samples of cooked *Rista* of about 50 g each was drawn separately from the four respective types of *Rista*. The samples so obtained were packaged in properly labeled LDPE bags and frozen stored at −20°C.

### mtDNA extraction

The chemicals utilized for mtDNA extraction from the test samples were lysis buffer, proteinase-K, TE buffer, phenol (tris saturated, pH 8.0), 10% sodium dodecyle sulfate (SDS), chloroform, isoamyl alcohol, isopropyl alcohol, ethanol, 3M sodium acetate (pH 5.5). The mtDNA from test samples was extracted as per the standard protocol described by Ausubel *et al*. [23] with some modifications. About 300 mg aliquot of the frozen test sample was cut into small pieces with a sterile scalpel and transferred to autoclaved porcelain mortar. The sample pieces were ground thoroughly by pestle with additions of liquid nitrogen. The test sample homogenate was transferred into a sterile 15 ml tube, and liquid nitrogen was allowed to evaporate. Lysis buffer - ST (0.5 ml) was added to the tube along with 5 µl proteinase K and 10% SDS (100 µl) to make final concentration of the latter to 2%. The homogenate was incubated for 12-16 h (overnight) at 55°C. At the end of incubation, the lysate was transferred to an autoclaved 15 ml centrifuge tube and equal volume 0.5 ml of tris saturated phenol (pH-8.0) was added and mixed gently for 10 min. The lysate was then centrifuged at 10,000 rpm and 15°C for 10 min. The supernatant was transferred to a 2 ml centrifuge tube and half the volume of tris saturated phenol:chloroform:isoamyl alcohol (25:24:1) was added and mixed gently for 10 min. It was centrifuged at 10.000 rpm and 15°C for 10 min. The supernatant was transferred into 2 ml centrifuge tube and equal volume of chloroform:isoamylalcohol (24:1) was added and mixed gently for 10 min and centrifuged at 10,000 rpm and 15°C for 10 min. The supernatant was collected into a 2 ml centrifuge tube followed by the addition of 1/10^th^ volume of 3M sodium acetate (pH 5.5) and equal volumes of isopropyl alcohol. The tubes were slowly swirled to precipitate the DNA which was then washed thrice with 70% ethanol and air dried and then dissolved in 200 µl volume autoclaved triple distilled water using properly marked 2 ml tubes. The DNA samples (stock solution) were stored at −20°C until further use. Quality, purity, and concentration of the extracted DNAs were checked by agarose gel electrophoresis and spectrophotometry.

### Primers for PCR

Various primers used in the present study were as per Zarringhabaie *et al*. [24] and are listed in Table-3. The primers were procured from M/s RFCL Ltd., New Delhi and were supplied in freeze-dried form and were stored at −20°C until their usage.

**Table-3:** Primers used in multiplex PCR.

Name	Primer type	Sequences (5’- 3’)	Size (bp)
Common	Reverse	TGTCCTCCAATTCATGTGAGTGT	-
Buffalo	Forward	TCCTCATTCTCATGCCCCTG	124
Cattle	Forward	TCCTTCCATTTATCATCATAGCAA	472
Sheep	Forward	TACCAACCTCCTTTCAGCAATT	585

PCR=Polymerase chain reaction

### PCR analysis

The multiplex PCR was carried out in a thermocycler using 0.2 ml thin wall PCR tubes. The 25 µl PCR reaction volume comprised 1.6 µl MgCl_2_ (1.5 mM), 0.5 µl M dNTP (0.2 mM), 4.5 µl of common reverse primer and 1 µl of each forward primer of sheep, cattle and buffalo (0.015 mM), 2 μl of template DNA (50 ng), 2.5 µl of ×10 PCR buffer, 0.06 µl Taq DNA polymerase (0.3 units) and remaining volumes (10.84 µl) of autoclaved triple distilled water. The optimized PCR protocol comprised initial denaturation for 3 min at 94°C, followed by 34 cycles of denaturation for 30 s at 94°C, annealing at 60°C for 45 s, extension at 72°C for 45 s and a final extension at 72°C for 10 min. The PCR products were electrophoresed at 85 V for 2 h in 2% agarose gels after the wells were charged with 5 µl of DNA preparations mixed with 1 μl of 6X gel loading buffer dye and viewed under ultraviolet transilluminator gel documentation after staining with ethidium bromide. The sizes of PCR products were determined in relation to a 100 bp DNA ladder.

## Results

**Figure-1:**
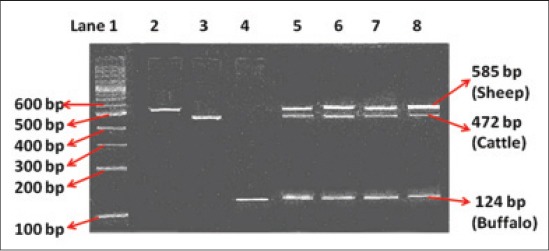
Species-specific multiplex polymerase chain reaction profile of *cyt b* gene fragments of sheep, cattle and buffalo from cooked *Rista*. Lane 1: DNA ladder, Lane 2: Pure mutton, Lane 3: Pure beef, Lane 4: Pure buffalo meat, Lane 5-8: Mixed meat (mutton:beef:buffalo meat in the ratio of 60:20:20, 80:10:10, 90:05:05 and 98:01:01, respectively).

The multiplex PCR profile of cooked pure and mixed meat *Rista* is present in Figure-1. The multiplex PCR amplified fragments exhibited the expected species-specific band patterns. The amplified bands of *cyt b* gene fragments were of the size of 124 bp, 472 bp and 585 bp for buffalo, cattle and sheep, respectively. For each mixed meat cooked *Rista* sample, three bands are present representing the meats of three species (sheep, cattle and buffalo) in the mixed meat of cooked *Rista*. In the case of pure meat *Rista* of mutton, beef and buffalo meat, only one band of the respective species is observed. It was possible to trace beef and buffalo meat in the cooked mutton *Rista* up to a level as low as 1%. In pure meat samples, band intensities where of similar intensity. In mixed meat samples, the band intensities for the cattle and buffalo *cyt b* gene fragments showed a progressive decrease with the decrease of their proportion from 20% to 1% in the cooked mutton *Rista*. The overall band intensities for sheep, cattle and buffalo *cyt b* gene fragments were lower due to DNA degradation during cooking. The multiplex PCRs for cooked *Rista* were repeated 3 times with 100% reproducibility. Based on above observations, a highly sensitive method for detection of mixed meats in cooked mutton *Rista* by multiplex PCR stands standardized.

## Discussion

The adulteration of meat species is a worldwide problem, which infringed food labeling laws, constitutes economic fraud, and raises ethical, religious and food safety concern [25]. Hence, detection of meat species by fast and accurate methods should routinely be carried out for the quality control as well as a public task to secure the food safety all over the world [26]. The speciation of cooked meat is difficult since the heat treatment during cooking causes extensive changes within the meat tissue [27]. In spite of these changes, short nucleic acid sequences are able to survive cooking processes which can be employed for the meat speciation purposes [28]. Hence, from the past two decades, significant efforts have been invested in the development of DNA-based meat speciation techniques. Nucleic acid-based analysis had widely used in many fields, and become more and more popular for differentiation and identification of feed or food adulterants [13]. The most recently DNA-based PCR assays are widely employed for differentiation and identification of species origin of meat and meat products [29-33], due to high heat stability and highly conserved nature of DNA [16], increases the probability of positive results even in highly fragmented DNA in highly processed meat products [34]. Ballin, 2010 [35] also has reported that while the presence and characteristics of proteins depend on the tissue type being analyzed, DNA exists and is identical in almost all cells, and the unique variability and diversity afforded by the genetic code permits the discrimination of even closely-related species. Detection method based on mtDNA can improve the sensitivity further because of the presence of thousands of copies of mtDNA per cell against just a few sets of genomic DNA. The mtDNA are easier to be extracted as they are located in the cytoplasm [6]. Genes to be targeted for amplification can be 12S, 16S and 18S rRNA, actin, cytochrome b, cytochrome oxidase-II, nicotinamide adenine dinucleotide dehydrogenase 5/6 and mtD-loop [36,26]. The variation of mitochondrial *cyt b* gene has been a rich source of phylogenetic inference in a wide range of animal species. The chances of mtDNA degradation under different meat processing conditions are lesser thus making it ideal for processed meat species identification [10]. Further, a higher copy number of mtDNA ensures a sufficiently large quantity of PCR product even in the case of samples undergoing intense DNA fragmentation [37].

The lowest detection level in the study was up to 1%. Detection of meat speciation at similar levels of addition in mixed meats has been reported by Rodriguez *et al*. [38]. Furthermore, there were progressive decreases of the levels of beef and buffalo meat in the mixed meat cooked product samples and, therefore, the concentration of beef and buffalo meat mtDNA (in the extracted mtDNA) also showed progressive decrease with the reduction in the level of the two mixed meats from 20% to 1%. As the band intensities and quality of mtDNA are correlated, therefore, the band intensities of beef and buffalo meat amplified DNA also showed a progressive decrease with the reduction of levels of these meats. These results on cooked *Rista* were in general conformity with the findings of Matsunaga *et al*. [8], Jain [39] and Sakalar and Abasiyanik [40]. The band intensities of beef and buffalo meat *cyt b* gene fragments in cooked *Rista* were lower due to DNA fragmentation during cooking. The heat treatment affects the quality of DNA has also been reported by Martinez and Man [41] and Hird *et al*. [42].

## Conclusion

The results of the present study clearly indicated that processing, the addition of cooking ingredients and cooking by traditional Kashmiri methods had no effect on detection of species of meat in the *Rista*. The present study supports the capability of mtDNA-based test to detect the meat falsification even at a lower level and is a herald to the fact that PCR is the method of choice for identifying meat species more efficiently and accurately even in highly processed Kashmiri cuisine products.

## Authors’ Contributions

This study is the part of M.V.Sc. thesis of the first author MMB, who carried out the research under the guidance of MS. IAM and HJ helped during the trial. SA and MAP helped in technical writing and revision of the article. All authors have read and approved the final version of the manuscript.
